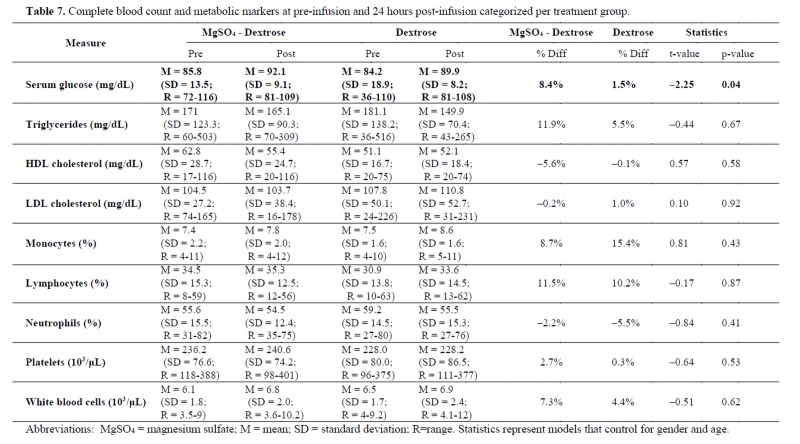# Intravenous infusion of magnesium sulfate is not associated with cardiovascular, liver, kidney, and metabolic toxicity in adults

**Published:** 2018-04-04

**Authors:** Elisa Karhu, Steven E Atlas, Jinrun Gao, Syed A. Mehdi, Dominique Musselman, Sharon Goldberg, Judi M. Woolger, Raul Corredor, Muhammad H. Abbas, Leopoldo Arosemena, Simone Caccamo, Ashar Farooqi, Janet Konefal, Laura Lantigua, Vanessa Padilla, Ammar Rasul, Eduard Tiozzo, Oscar L. Higuera, Andrea Fiallo, John E. Lewis

**Affiliations:** ^1^ Departments of Medicine; ^3^ Psychiatry and Behavioral Sciences; ^5^ Family Medicine and Community Health, University of Miami Miller School of Medicine, Miami, FL, USA;; ^2^ Barclay's, Inc., Wilmington, DE, USA;; ^4^ Glow Health PA, Bay Harbor Islands, FL, USA

**Keywords:** MgSO4, clinical pharmacology, drug toxicity

## Abstract

**Background:**

Magnesium (Mg) deficiency contributes to the pathophysiology of numerous diseases. The therapeutic use of Mg has steadily increased over time. The increased in-hospital use of intravenous (IV) magnesium sulfate (MgSO4) warrants more extensive investigation regarding the safety of the therapy. The aim of this study was to determine the safety of IV MgSO4 infusion on cardiovascular, liver, kidney, and metabolic markers in adults.

**Methods:**

Twelve volunteers were randomized to one of two cross-over conditions: (a) IV infusion of MgSO4 in 5% dextrose followed by IV infusion of 5% dextrose 1 week later or (b) IV infusion of 5% dextrose followed by IV infusion of MgSO4 in 5% dextrose 1 week later. An electrocardiogram was recorded continuously during the infusions. Blood was drawn pre- and post-infusion for blood count (high-density lipoprotein cholesterol, low-density lipoprotein cholesterol, and triglycerides). **Results:** Serum Mg increased from pre- to post-infusion in the MgSO4 + 5% dextrose group (p < 0.0001). The QRS interval length increased from pre- to post-infusion in the MgSO4 + 5% dextrose group (p < 0.04). Additionally, serum glucose concentration increased in the MgSO4 + 5% dextrose group (p = 0.04). These significant findings were modeled with gender and age as covariates. No other significant differences were found.

**Conclusions:**

The administration of IV infusion of MgSO4 (4 g/100 mL) in 5% dextrose over a 4-hour treatment period poses no significant deleterious effects on cardiovascular, liver, kidney, or metabolic function.

**Relevance for patients:**

IV infusion of MgSO4 may be used for certain treatment indications without significant concern for systemic or organ toxicity.

## 1. Introduction

Magnesium (Mg) is the fourth most abundant mineral in the body, acting as a cofactor in more than 300 enzymatic reactions and performing an important role in adenosine triphosphate metabolism [1-3]. Mg is required for protein synthesis, reproduction, and DNA and RNA synthesis. Mg is also crucial in regulating muscular contractions, cardiac excitability, blood pressure, vasomotor tone, insulin metabolism, nerve transmission, and neuromuscular conduction [1-3].The normal serum magnesium concentration in humans is 1.8 to 2.3 mg/dL [4]. Due to the many functions of Mg in the human body, it plays an important role in the prevention and treatment of many diseases.

Mg deficiency contributes to the pathophysiology of numerous diseases including hypertension, ischemic heart disease, arrhythmias, preeclampsia, asthma, and critical illness [5], and the therapeutic value of Mg for different conditions is under investigation in multiple trials. To date, intravenous (IV) magnesium sulfate (MgSO4) has been widely used in the treatment of preeclampsia and premature labor [6] and torsades de pointes [5]. More recently, we investigated the effect of IV MgSO4 infusion in adults with treatment-resistant depression and found modest improvement in depressive symptoms that were correlated with increases in serum Mg [7]. Despite the increasing therapeutic use of Mg, indications for the measurement of Mg levels and the treatment of hypomagnesemia with IV MgSO4 are not well-defined in hospitalized patients. Increases in the in-hospital use of Mg are also not clearly explained by medical indications [8].

In light of the increased use of Mg in the treatment of a variety of conditions, the safety of this therapy warrants further investigation. Although electrocardiographic (ECG) and electrophysiological effects of IV Mg infusion have been studied in experimental animals [9], human studies remain limited. A few studies have examined the effects of IV MgSO4 on electrolytes [10-12], cardiac conduction and refractoriness [10], cardiac hemodynamics [6,13], and colloid osmotic pressure changes [14] (Table 1). The purpose of the present study was to determine the safety of IV MgSO4 infusion on cardiovascular, liver, kidney, and metabolic parameters in adults.

## 2. Materials and Methods

### 2.1 Study participants

The study was conducted with the approval of the University of Miami Institutional Review Board for human subject research (protocol no. 20110168). Each subject signed informed consent and Health Insurance Portability and Accountability Act forms prior to study entry. Potential participants (n = 199) were identified through referrals from offices at the University of Miami School of Medicine, the Department of Psychiatry and Behavioral Sciences clinics, Center for Complementary and Integrative Medicine, advertisements around the University of Miami campus, from clinicaltrials.gov, and from Life Extension Foundation from October 2011 to December 2014. Twenty-two subjects were enrolled for the baseline screening, 13 subjects were eligible after baseline screening, and 12 participants were enrolled and randomly assigned to one of the two study conditions(Table 2). The current study was performed as part of a clinical trial that evaluated the effect of IV MgSO4 infusion on depressive symptoms in adults with treatment-resistant depression [7].

### 2.2 Inclusion and exclusion criteria

Inclusion criteria were: (a) age 21-70 years; (b) major depressive disorder diagnosis according to DSM-IV-TR criteria and treatment-resistant depression defined as failure of clinical improvement after 6 weeks with an approved,clinically adequate dose of a selective serotonin reuptake inhibitor (SSRI), a serotonin-norepinephrine reuptake inhibitor (SNRI), or a selective noradrenaline reuptake inhibitor (NRI); and (c) if currently taking an SSRI, SNRI,NRI, aripiprazole, quetiapine, risperidone, bupropion, or a TCA for more than 90 days, the participant must have maintained the same dose for the past 90 days prior to study enrollment. Exclusion criteria were: (a) a baseline serum Mg level >2.6 mg/dL (normal range 1.6-2.6 mg/dL); (b)currently enrolled (or in the last 30 days) in another research trial for investigative nutritional or other therapies thought to have an impact on depression; (c) currently taking an oral medication or nutritional supplement containing more than 100% RDA of Mg (for women over age 31, 320 mg/day and for men over age 31, 420 mg/day); (d) diagnosed with any medical condition that would preclude participation in the study; (e) taking oral digoxin, penicillamine, any antibiotic,or any other oral psychotropic medication (other than aripiprazole, quetiapine, or risperidone) for any indication,except sedatives for sleep, in addition to the SSRI, SNRI, or NRI in the course of treatment for depression; (f) pregnant,planning to become pregnant, currently breastfeeding, or unwilling to avoid pregnancy; (g) systolic blood pressure of >160 mm Hg or diastolic blood pressure of>90 mm Hg; or (h) any of the following abnormal laboratory test values: bilirubin >2x upper normal limit;aspartate aminotransferase and alanine aminotransferase >2x upper normal limit; serum creatinine >1.5 mg/dL; blood glucose <80 mg/dL or >110 mg/dL; serum calcium (Ca) level of <8.6 mg/dL; or triglycerides >200 mg/dL.

### 2.3 Screening

Study staff conducted a preliminary screening to determine whether the potential participant met criteria to take part in the study. If the preliminary screening was acceptable, the participant was scheduled for informed consent and study protocol procedures.

**Table 1.**
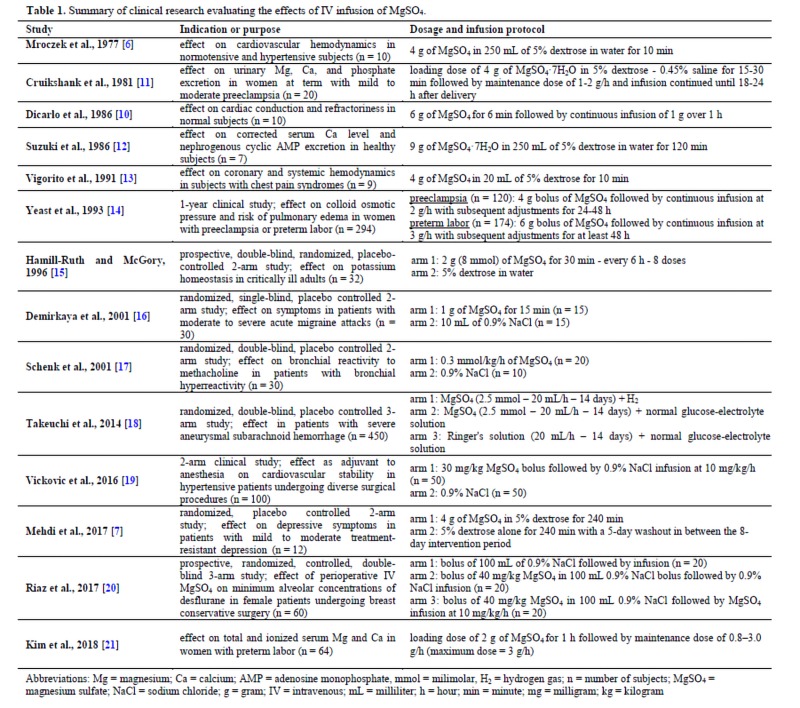
Summary of clinical research evaluating the effects of IV infusion of MgSO4.

### 2.4 Randomization

After screening and baseline measurements, participants were randomly assigned to one of two conditions: (a) IV infusion of MgSO4 in 5% dextrose followed by IV infusion of 5% dextrose 1 week later (Treatment A) or (b) IV infusion of 5% dextrose followed by IV infusion of MgSO4 in 5% dextrose 1 week later (Treatment B). Treatment assignment was done with a table of random permutations, which balanced the number of participants in each group, by the University of Miami research pharmacy. All subjects and investigators were blinded to treatment condition and remained blinded until after data analysis. Only one staff member at the research pharmacy knew the assignment. Infusion materials were provided by the study sponsor (Life Extension Foundation, Ft. Lauderdale, FL, USA).

### 2.5 Intervention schedule

Participants were scheduled for assessments and/ or treatments at baseline and on days 1, 2, 7, and 8. Each participant was required to fast prior to and for the 4 hours during each IV infusion on days 1 and 7.

### 2.6 Treatment

An MgS0_4_ injection USP solution (50% in 5 mL water at 0.5 g/mL) was diluted in 100 mL of 5% dextrose injection USP (https://www.drugs.com/pro/magnesium-sulfate.html). The 4 g (32.58 mEq Mg^2+^) dose of MgS0_4_ in 5% dextrose or 5% dextrose alone was administered through an IV via an infusion pump over 240 min (25 mL/hour) from a 100 mL bag. The IV line was not used to inject any other medications. Based upon the existing literature, the recommended dosage for Mg deficiency is 5 g (approximately 40 mEq Mg^2+^) and can be added to 1 L of 5% dextrose USP for slow IV infusion over a 3-hour period. Thus, the amount of Mg delivered was well within acceptable limits and not supra-physiological.

### 2.7 Baseline

At baseline, a health history form was completed, which elicited allergy information, medical and surgical histories, concomitant or recently taken medications, over-the-counter non-prescription products, nutritional supplements, and investigational products. Vital signs and ECG were assessed. Participants also underwent a full physical examination to verify eligibility and had fasting blood drawn. A serum or urine pregnancy test for female participants of childbearing potential was also performed.

### 2.8 Days 1 and 7

At each time point, participants had their fasting blood and urine specimens tested for items in the laboratory assessments listed below. Assessments included basic vital signs taken at pre-dose, 15 min, 30 min, 45 min, 60 min, 90 min, 120 min, 150 min, 180 min, 210 min, and 240 min. An ECG report was generated at pre-dose, completion of infusion, and 24 hours after infusion completion. An accountability was performed of the study product, and any deviations were noted. Participants had their fasting blood and urine specimens tested for items listed in the assessment section below.

### 2.9 Days 2 and 8

The participants underwent a brief physical exam and were interviewed to capture any adverse effects. The participants had basic vital signs taken. Fasting blood was collected for analysis as described below. A serum or urine pregnancy test was also performed on day 8 for female participants of childbearing potential. After finishing the procedures for day 8, the participants were discharged from the study.

### 2.10 Outcomes and assessments

All participants completed an extensive socio-demographics and medical history questionnaire and reported their list of medications at baseline. At the baseline visit, complete blood count (CBC) and clinical chemistry (serum and urine Mg, high-density lipoprotein (HDL) and low-density lipoprotein (LDL) cholesterol, and triglycerides), and urinalysis were assessed. During the IV infusion phase (days 1 and 2 and days 7 and 8), the following assessments were performed: (a) ECG, CBC/ chemistry, HDL and LDL cholesterol, triglycerides, and serum Mg at pre-dose on days 1 and 7, at infusion completion on days 1 and 7, and 24 h after infusion completion on days 2 and 8, and (b) a pregnancy test via serum or urine was conducted at baseline and on day 8.

### 2.11 Intervention protocol

Participants had a staff member with them at all times to ensure compliance. Subjects were not advised to modify eating or physical activity habits or prescription medication use during the protocol. In addition, they were instructed not to consume any dietary supplement containing Mg during the trial. Subjects listed all dietary supplements taken on the health history form, and products were reviewed to ensure none of these nutrients was consumed during the course of the trial.

### 2.12 Statistical analysis

Data were analyzed using SAS 9.3 (SAS, Durham, NC, USA). We used the general linear model to assess changes over the course of the intervention (administration of the MgS0_4_) among the primary outcome variables, including the ECG values, heart rate, blood pressure, and blood and urine tests. For each outcome variable, we tested the effect of administering the MgS0_4_ (MgS0_4 _+ 5% dextrose vs. 5% dextrose) on pre-post percent change. The two groups, MgS0_4_ + 5% dextrose and 5% dextrose, were clustered for analysis. The general linear models were performed two ways: (a) controlling for gender and age and (b) not controlling for gender and age. The reported results are with controlling for gender and age only, since that method is theoretically sound, empirically supported, and to report the other models would be redundant. The relevant model statistics, t-test value, degrees of freedom in brackets, and corresponding p level are listed. The higher the t-test value (and the lower the p level), the more likely that a difference in an outcome variable is significant. The criterion for statistical significance was a = 0.05. A p value of <0.05 was considered statistically significant.

## 3. Results

### 3.1 Safety and tolerability

During the entire study period, one subject had an elevated blood pressure response during the infusion of MgS0_4_, but it was apparently due to not taking her hypertension medication the morning of the infusion. Once her medication was administered, her blood pressure normalized. No other adverse events were reported.

### 3.2 Cardiovascular function

As noted in Table 3, a significant difference was observed in the MgSCM + 5% dextrose vs. 5% dextrose alone QRS interval length change from pre- to post-infusion (t = -2.25 [1], p = 0.04), controlling for age and gender. The QRS interval length increased from 83.6 + 19.6 to 87.1 + 18.9 milliseconds (msec) pre- to post-infusion in the MgSCM + 5% dextrose group and decreased from 85.3 + 17.9 to 83.5 + 17.1 msec in the 5% dextrose alone group. No other significant differences were noted in the cardiovascular markers.

### 3.3 Liver enzymes

No significant differences were found in the liver enzymes shown in Table 4.

### 3.4 Kidney function

No significant differences were found in the kidney function markers shown in Table 5.

### 3.5 Serum electrolytes

As seen in Table 6, a significant difference was observed in the MgS04 + 5% dextrose vs. 5% dextrose alone serum Mg percent change from pre- to post-infusion (t = 10.04 [1], p < 0.0001), controlling for age and gender. Serum Mg increased from 2.0 + 0.2 to 3.2 + 0.4 mg/dL pre- to post-infusion in the MgS04 + 5% dextrose group and decreased from 2.0 + 0.3 to 1.9 + 0.1 mg/dL in the 5% dextrose alone group. No other significant differences in electrolyte concentrations were noted.

### 3.6 Complete blood count and metabolic markers

As seen in Table 3, a significant difference was observed for the percent change in serum glucose from pre- to post-infusion (t = -2.25 [1], p = 0.04), controlling for age and gender. Serum glucose increased from 85.8 + 13.5 to 92.1 + 9.1 mg/dL pre- to post-infusion in the MgS04 + 5% dextrose group and also increased from 84.2 + 18.9 to 89.9 + 8.2 mg/dL in the 5% dextrose alone group. No other significant differences were found in metabolic or CBC markers.

## 4. Discussion

The aim of the current study was to determine the safety of IV MgSCM infusion in adults. Based on our findings, the administration of IV MgS04 (4 g/100 mL) in 5% dextrose over 4 hours poses no deleterious effects on cardiovascular, liver, kidney, and metabolic function. In our study, serum Mg concentrations increased significantly in the MgS04 treatment group from pre- to post-infusion, which was consistent with previous studies (Table 1) [6,10-13].

Likewise, we observed no significant differences in liver enzymes and kidney function markers in the current study. A small increase in serum glucose was noted in the MgSO4 infusion group when corrected for age and sex, but the significance was lost in the uncorrected model. The slight increase in serum glucose may have been caused by the infused dextrose, which has previously been shown to elicit a significantAn increase in the QRS interval from 83.6 + 19.6 to 87.1 + 18.9 msec pre- to post-infusion in the MgS04 plus 5% dextrose group was noted, but no other significant differences were seen in cardiovascular (ECG) markers. A recent prospective study suggested increased QRS interval to be predictive of sudden cardiac death with a 2.5-fold risk elevation in subjects with QRS interval >110 msec compared to QRS interval <96 msec [22]. In light of these prior findings, the QRS interval length increase noted in our study does not appear to be clinically significant, as it is still within the range of normal physiological variation (69-109 msec) [22]. Current evidence also indicates that drug-induced changes in the QRS interval observed in healthy subjects do not hold the same clinical and prognostic significance as the same findings in patients with existing cardiovascular disease [22]. Another study by DiCarlo et al. previously showed no significant change in QRS interval or heart rate with infusion of 6 g IV MgS04 over 6 minutes [10]. In contrast, a study by Vigorito et al. noted an increase in heart rate after MgS04 infusion [13], while in our study a small non-significant increase in heart rate (69.3 + 12.0 to 73.0 + 12.3, p = 0.10) was found. However, the infusion rate of 4 g MgS04 in 10 minutes used by Vigorito et al. was much faster than the rate used in our study. Additionally, no significant change occurred in mean arterial pressure during our study, which is supported by results from Mroczek et al., who also noted no change in mean arterial pressure in normotensive subjects and a transient fall in mean arterial pressure in hypertensive subjects [6].short-term increase in serum glucose [23]. In our study, 5 g of dextrose was infused, which provided 17 kcal per dose. No other significant changes in electrolyte concentrations were observed. A significant decrease in serum Ca during MgSO4 infusion was previously noted by Cruikshank et al. [11] but was not observed in our study. Once again, the infusion rate used by Cruikshank et al. was much faster at 4 g in 15-30 minutes compared to our protocol. A similar decrease in serum Ca was noted by Suzuki et al. [12], who also utilized a faster infusion protocol of 9 ?1.2 g MgSO4 over 2 hours.

## 5. Conclusions

Taken together, our data on cardiovascular, liver enzyme, kidney, and metabolic function markers indicate that MgSO4 can be safely infused by the IV route. These findings add to the current literature that has shown IV MgSO4 infusion to be a safe therapeutic modality. The results should be valuable for encouraging the development of studies to investigate new IV MgSO4 treatment paradigms for a variety of clinical disorders.

**Table:**
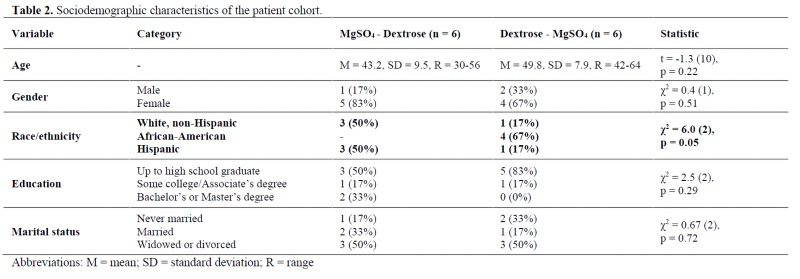


**Table:**
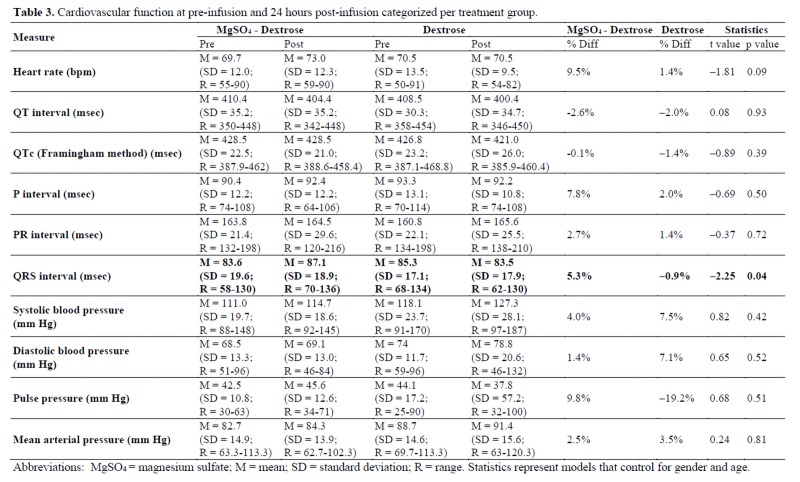


**Table:**
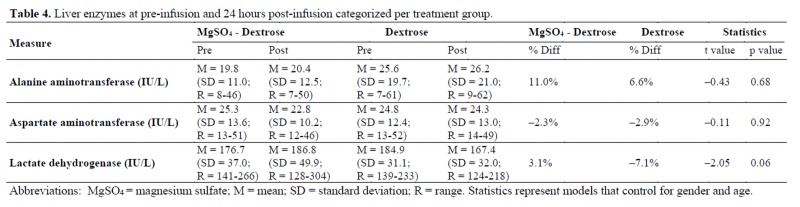


**Table:**
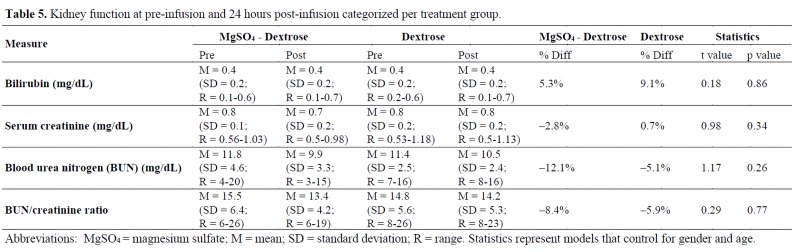


**Table:**
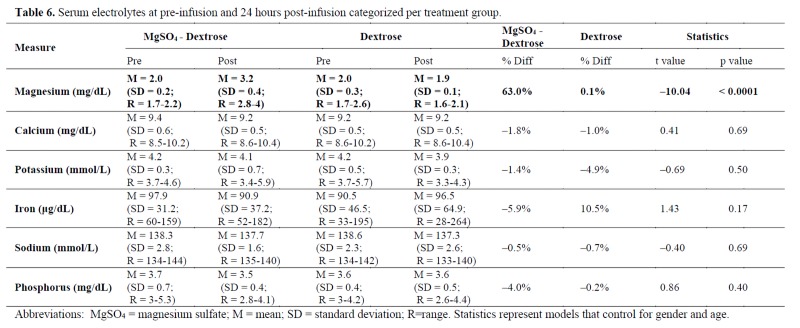


**Table:**